# Metal Nanoparticles Obtained by Green Hydrothermal and Solvothermal Synthesis: Characterization, Biopolymer Incorporation, and Antifungal Evaluation Against *Pseudocercospora fijiensis*

**DOI:** 10.3390/nano15050379

**Published:** 2025-02-28

**Authors:** Tania Caguana, Christian Cruzat, David Herrera, Denisse Peña, Valeria Arévalo, Mayra Vera, Pablo Chong, Néstor Novoa, Ramón Arrué, Eulalia Vanegas

**Affiliations:** 1 Master Program in Environmental Sciences, Faculty of Chemical Sciences, Eco Campus Balzay, University of Cuenca, Cuenca 010207, Ecuador; tania.caguana@ucuenca.edu.ec; 2 N@NO-CEA Group, Center for Environmental Studies, Department of Applied Chemistry and Production Systems, Faculty of Chemical Sciences, University of Cuenca, Cuenca 010203, Ecuador; david.herrerar@ucuenca.edu.ec (D.H.); denisse.pena@ucuenca.edu.ec (D.P.); valeriaf.arevalo@ucuenca.edu.ec (V.A.); eulalia.vanegas@ucuenca.edu.ec (E.V.); 3 TECNO-CEA Group, Center for Environmental Studies, Department of Applied Chemistry and Production Systems, Faculty of Chemical Sciences, University of Cuenca, Cuenca 010203, Ecuador; mayra.vera@ucuenca.edu.ec; 4 Centro de Investigaciones Biotecnológicas del Ecuador, Laboratorio de Biología Molecular, Campus Gustavo Galindo, ESPOL Polytechnic University, Km 30.5 Vía Perimetral, Guayaquil 090902, Ecuador; pachong@espol.edu.ec; 5 Laboratorio de Química Inorgánica y Organometálica, Departamento de Química Analítica e Inorgánica, Facultad de Ciencias Químicas, Universidad de Concepción, Edmundo Larenas 129, Casilla 160-C, Concepción 4070386, Chile; nenovoa@udec.cl; 6 Facultad de Medicina y Ciencia, Departamento de Ciencias Biológicas y Químicas, Universidad San Sebastian, Lientur 1457, Concepción 4070386, Chile; ramon.arrue@uss.cl

**Keywords:** copper nanoparticles, cobalt nanoparticles, green hydrothermal synthesis, black sigatoka disease, *Pseudocercospora fijiensis*

## Abstract

Nanoparticles (NPs) have generated significant interest in various fields due to the unique properties that materials exhibit at the nanoscale. This study presents a comparative analysis of copper nanoparticles (Cu-NPs) and cobalt nanoparticles (Co-NPs) synthesized via conventional solvothermal and green hydrothermal synthesis using ethylene glycol and *Medicago sativa* extract, respectively. The conventional solvothermal synthesis showed higher efficiency for both Cu-NPs and Co-NPs with yields of 32.5% and 26.7%, respectively. Characterization through UV–visible spectroscopy (UV–vis), Fourier-transform infrared spectroscopy (FTIR) and atomic force microscopy (AFM) revealed that while solvothermal synthesis produced larger particles (76.5 nm for Cu-NPs, 86.8 nm for Co-NPs), the green hydrothermal method yielded smaller particles (53.8 nm for Cu-NPs, 67.7 nm for Co-NPs) with better control over particle size distribution and spherical morphology, showing minimal agglomeration. UV–vis confirmed metal oxide formation, while FTIR showed complex patterns in NPs (green hydrothermal), indicating plant extract compounds. Antifungal evaluation against *Pseudocercospora fijiensis* showed complete inhibition at 2000 ppm for both NP types, with no mycelial growth after 30 days. When integrated into chitosan, solvothermal NPs produced rougher surfaces, and scanning electron microscope (SEM) confirmed the presence of copper and cobalt in the nanocomposites. This study provides insights into the synthesis of nanoparticles using an environmentally friendly process and their microbiological applications for future use in organic agriculture.

## 1. Introduction

Nanotechnology is the study and application of physicochemical phenomena that manifest and intensify in materials when working at the nanometric scale [1,2]. Nanometric materials exhibit enhanced characteristics including increased surface area [3], and magnetic properties such as superparamagnetism are present [4]. Nanomaterials also exhibit higher catalytic activity in chemical reactions due to the greater proportion of atoms at the corners and edges compared to the center of the nanoparticle [5]. This promotes the controlled fabrication of materials in different 2D and 3D nanostructures, leading to improved biosensor capabilities, biodetection, better drug release control [6,7,8], and low-toxicity [9]. Due to their size, these compounds can be applied in small quantities, making them environmentally friendly and cost-effective [10]. The transition metals, including Cu and Co, have received significant attention in recent years due to their catalytic, magnetic [11,12], optical [13,14], and biological properties [15,16]. For instance, copper nanoparticles (Cu-NPs) have shown potential industrial applications as gas sensors or in catalytic processes [17,18], as well as in high-temperature superconductors and solar cells [19,20]. These materials have also been used to replace silver, gold, or platinum nanoparticles in fields such as thermal conductors and microelectronics [21,22]. Similarly, cobalt nanoparticles (Co-NPs) have broad applications in areas such as microelectronics, biomedicine, and catalysis [23,24]. While Cu-NPs are widely studied due to their cost-effectiveness and availability [25,26], Co-NPs have emerged as an alternative, especially in agricultural applications, where they do not induce toxicity [27]. For example, it has been demonstrated that the use of Co-NPs is effective in aiding the growth of the *Arabidopsis* plant, regulating chlorophyll synthesis. Additionally, Co-NPs acted as antibacterial agents in the same plant [28]. Nanoparticles (NPs) can be synthesized using two techniques: bottom-up and top-down. The bottom-up technique involves the self-assembly of atoms into nuclei to form nanometric materials, using chemical and biological methods. The top-down technique breaks down large materials into small particles through size reduction, employing physical and chemical methods [29,30,31]. Physical methods reduce particle size through grinding, laser pulverization, and evaporation–condensation. Chemical methods include reduction, microemulsion, synthesis in humid environments, spray pyrolysis, precipitation, and microwave-assisted combustion. Furthermore, biological methods, through the use of microorganisms and green synthesis, employ compounds extracted from plants [31]; these are considered the most economical, and ecological option at present [32]. The synthesis method influences the final characteristics of the NPs, affecting their size, shape, and stability [33]. In this regard, the use of electromagnetic radiation has proven to be an important method for obtaining NPs with the desired size and shape [34,35]. It has been shown that by carefully adjusting the electromagnetic source, both the size and shape of the NPs can be controlled [36]. These characteristics are important for their effectiveness in various applications, including plant disease control, where they can induce oxidative stress, modifying cell membranes and organelles, and inhibiting fungal growth and spore germination [37].

One of the most widely used chemical methods for the synthesis of NPs is the solvothermal technique. This offers reliable and reproducible protocols to achieve precise morphological control [38]. This process involves the use of solvents and requires high temperatures and pressures in specific equipment [39]. There is also hydrothermal synthesis, which refers specifically to chemical reactions carried out in aqueous media at elevated temperatures and pressures above the normal boiling point of water, typically using sealed reactors that allow precise control of reaction conditions [40,41].

Green synthesis or biosynthesis of NPs uses active principles from plants, fungi, or bacteria, offering environmental and economic advantages over physical and chemical methods [42]. This bottom-up process involves reduction/oxidation reactions, where microbial enzymes or plant phytochemicals reduce metal compounds to NPs [43]. Shedbalkar et al. point out that biosynthetic NPs are more stable, morphologically controllable, and scalable [44]. A recent and noteworthy review on the green synthesis of nanoparticles highlights the key role of active components—such as flavonoids, saponins, alkaloids, tannins, and plant phenolics—in reducing metal salts to stable nanoparticles [45]. Studies have demonstrated successful synthesis of NPs using various plant extracts, reaching controlled morphology and enhanced stability [46,47]. Research highlights include the use of *Mentha pulegium*, *Eucalyptus globulus*, and *M. calabura* for synthesis, as well as ascorbic acid as a reducer, showcasing applications in plant disease control and growth enhancement [48,49]. In particular, *Medicago sativa* (alfalfa) presents an excellent candidate for green synthesis due to its rich content of bioactive secondary metabolites, including terpenoids, polyphenols, and flavonoids [50,51]. The integration of green principles with hydrothermal methodology creates a sustainable approach that combines the morphological control of hydrothermal synthesis with the eco-friendly characteristics of green synthesis [52,53,54].

On the other hand, the incorporation of NPs into biopolymer matrices is an important strategy to improve their stability and application [55]. Natural biopolymers such as chitosan (CS) are used to achieve this objective. CS, a natural biopolymer derived from chitin, has garnered significant interest due to its biocompatibility, biodegradability, and ability to form stable matrices with metal-NPs [56]. In this context, composite characterization is necessary to obtain information about the distribution of NPs within the matrix and the nature of polymer–metal interactions. A promising application of these NPs is their use in the agricultural area, where there are challenges, such as the biological control of black Sigatoka disease, caused by the fungus *Pseudocercospora fijiensis*, which can reduce banana crop yields by up to 80% [57,58]. These crops are extremely important for Ecuador, a country that leads the export of this fruit and benefits 2.5 million people by generating income and employment [59]. While traditional chemical control methods remain prevalent, environmental concerns and pathogen resistance have driven research toward alternative solutions [60], with characterized and stabilized NPs that offer a reliable and effective long-term strategy [61].

This research aims to conduct a comparative study of Cu-NPs and Co-NPs synthesized through solvothermal and green hydrothermal synthesis methods using alfalfa extract. The investigation focuses on comprehensive physicochemical characterization using UV–vis, AFM, SEM, and FTIR analysis to understand how different synthesis routes influence NP properties and their antifungal potential against *Pseudocercospora fijiensis*. SEM was employed to characterize the nanocomposite structure and confirm the successful incorporation of metal NPs. This comparative approach will provide valuable insights into the relationships between synthesis methods, NPs characteristics, and their potential agricultural applications.

## 2. Materials and Methods

### 2.1. Materials

This study used copper (II) nitrate hemipentahydrate (Cu(NO_3_)_2_·2.5H_2_O—98%—Sigma Aldrich, St. Louis, MO, USA) and cobalt (II) nitrate hexahydrate (Co(NO_3_)_2_·6H_2_O—97%—Sigma Aldrich) as precursor agents.

Anhydrous ethylene glycol (99.8%—Sigma Aldrich) as reductor agent. For bioassays, strain 630 of the fungus *Pseudocercospora fijiensis* was used and Potato dextrose agar (PDA) as a culture medium

### 2.2. Preparation of Medicago Sativa Extract

*Medicago sativa* leaves were collected, washed with distilled water, shade-dried, and crushed. Subsequently, 40 g of the crushed material and 400 mL of distilled water were heated at 60 °C for 3 h. The resulting solution was filtered using a funnel and Whatman filter paper. Once filtered, it was stored in an amber bottle at 4 °C until it was used.

### 2.3. Synthesis of NPs

**Figure 1:**
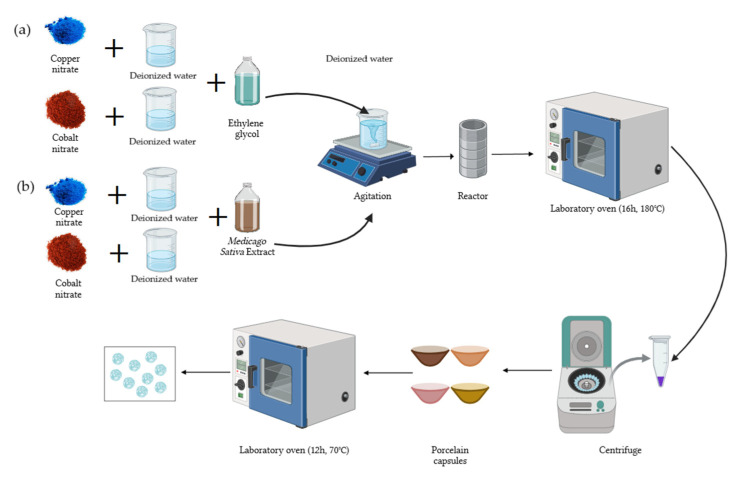
Schematic of the NP synthesis process. (**a**) Solvothermal synthesis; (**b**) green hydrothermal synthesis.

For the green hydrothermal method of the synthesis of copper-NPs and cobalt-NPs, the precursor amount was determined based on the final solution volume, representing 5% of it (Figure 1). Reactors of 120 mL and 45 mL were used for Cu-NPs and Co-NPs, respectively. First, 0.05 moles (13.96 g) of Cu(NO_3_)_2_·2.5H_2_O and 0.01 moles (2.85 g) of Co(NO_3_)_2_·6H_2_O were weighed. Copper nitrate was then dissolved in 45 mL of deionized water and cobalt nitrate in 10 mL of deionized water. The solutions were made up to 120 mL with *Medicago sativa* extract for Cu and up to 30 mL for Co. The mixture was stirred on a stirring plate for 20 min. Subsequently, the solutions were placed in the reactor, which was introduced into the oven at 180 °C for 16 h. Washes were performed with deionized water and ethanol until a neutral pH was obtained. The product was dried in the oven at 70 °C for 12 h. The obtained powder was weighed and stored.

The solvothermal synthesis was the same as in the previous section, but ethylene glycol was used instead of *Medicago sativa* extract.

### 2.4. Characterization of NPs

UV–vis analysis: The properties of Cu-NPs and Co-NPs synthesized via green hydrothermal and solvothermal synthesis were analyzed using UV–vis spectroscopy (Genesys, Madison, WI, USA) to confirm the synthesis of NPs. Solutions with a concentration of 1 mg/mL were prepared. Ethanol and ethylene glycol were used as blank solutions for green hydrothermal synthesis and solvothermal NPs, respectively. For alfalfa extract analysis, distilled water was used as a blank. Each solution was rigorously shaken to ensure homogeneity before being measured by the Genesys 10S UV–vis spectrophotometer (Madison, WI, USA).

Determination of the pH of the point of zero charge (pH_pzc_): This measure indicates the pH value at which the surface of the NPs has a net charge of zero. Forty milligrams of the previously synthesized NPs was weighed. From this amount, 5 mg were distributed in eight Erlenmeyer flasks. Then, 50 mL of deionized water was added to each flask. Subsequently, the initial pH of each suspension was measured and adjusted using 0.1 M HCl or NaOH solutions to obtain pH values of 2, 3, 4, 5, 6, 7, 9, and 11. Once the pH was adjusted, the flasks were placed in an orbital shaker (Shaker Thermo Scientific Maxq 4000, Waltham, MA, USA) at a speed of 250 rpm and a constant temperature of 25 °C for 24 h. After this period, the pH of each solution was measured again to evaluate the variation and determine the point of zero charge.

AFM analysis: NP characterization was conducted using the NX-10 Atomic Force Microscopy equipment manufactured by Park System (Suwon, Republic of Korea). The experiments were performed in non-contact mode with an NCHR model cantilever (NCHR NanoWorld Group, Neuchâtel, Switzerland). A glass slide was used as a substrate, previously treated with a sulfochromic solution to remove contaminants and obtain a suitable surface for sample deposition. Subsequently, the slides were washed with ethanol and dried in an oven for 2 h to ensure the elimination of any trace of moisture or solvent. Then, 5 mg of NPs was prepared and dissolved in 1 mL of isopropyl alcohol. The resulting suspension was subjected to a sonication process for 30 min to ensure adequate dispersion of the NPs in the solvent and prevent the formation of aggregates. Once dispersed, 20 µL of the NP solution was deposited on the treated glass substrate. The samples were dried using nitrogen (N_2_). After substrate preparation, the samples were analyzed using AFM, obtaining high-resolution images that allowed the evaluation of morphology, topography, and distribution of the NPs. For the analysis of the size and average diameter of Cu-NPs and Co-NPs, the software XEI 4.3.4 Build 22 developed by Park Systems Corp. was used. Within this software, the grain detection method tool was employed, which allowed for the identification of NPs present in the sample.

SEM and X-ray energy dispersive spectroscopy (EDS) analysis: Nanocomposite characterization was carried out using a Zeiss Gemini SEM 360 field emission scanning electron microscope (FE-SEM, Zeiss, Oberkochen, Germany). Samples were deposited on conductive sample holders and coated with a thin layer of gold by sputtering to improve conductivity and optimize image quality. An accelerating voltage (EHT) of 5.00 kV was employed and images were acquired at magnifications in the range of 5000× to 20,000×, ensuring adequate resolution for morphological and structural characterization of the NPs. EDS analysis was performed in mapping mode, allowing the identification and quantification of the elements present in the samples. To minimize loading effects, the vacuum and detection conditions were adjusted according to the properties of each sample.

FTIR analysis: This was performed using the Thermo Scientific Nicolet Summit Pro FTIR spectrometer (Thermo, Madison, WI, USA), running a total of 16 scans per sample to obtain the corresponding spectrum. For each test, a small amount of NPs (fine powder) was placed on the spectrometer platform, ensuring uniform dispersion. Three replicates were performed per sample to verify the reproducibility of the results and ensure the accuracy of the analysis.

### 2.5. Bioassays

The methodology used for conducting bioassays was known as the mycelial growth inhibition assay. NPs at a concentration of 2000 parts per million were used, dissolved in PDA medium, specifically for fungal culture. The concentration was selected based on previous studies that have demonstrated the effectiveness of NPs at similar concentrations against various phytopathogenic fungi [62,63,64]. In 25 mL of sterile water, *Pseudocercospora fijiensis* spores were suspended, and hyphae were dissolved using a vortex for one minute, followed by a one-minute rest, then the process was repeated once more. Plating was performed in a laminar flow cabinet. A 500 µL aliquot of the fungal suspension was taken and placed on plates with PDA medium, both for control and NPs. With a Drigalski loop, the fungus was spread uniformly over the medium, and the plates were properly sealed. Finally, the plates were stored at a controlled temperature (28 °C) for the proper growth of the *Pseudocercospora fijiensis* fungus. Four replicates of each sample were made and evaluated over a period of 30 days. Petri dishes of 90 × 15 mm were used for the experiment. ImageJ 1.54g software was employed to determine mycelial growth.

### 2.6. CS-NP Nanocomposite

**Figure 2:**
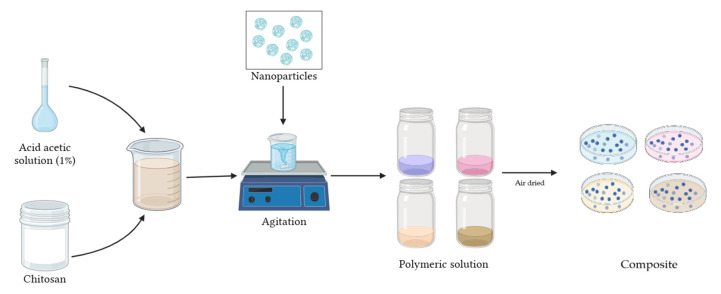
Schematic of CS-NP nanocomposite preparation.

In 150 mL acetic acid solution (1% *w*/*w*), 3 g of medium molecular weight CS were dissolved, stirring until a homogeneous mixture was obtained. To this mixture, 150 mg (5%) of NPs were added, stirring again until homogenized (Figure 2). Thus, the CS polymeric solution with Cu-NPs presented a grayish coloration and the CS polymeric solution with Co-NPs presented a light pink color. Meanwhile, the polymeric solution formed by NPs synthesized by the green hydrothermal synthesis presented light green color (Cu-NPs) and dark gray (Co-NPs). The polymeric solution was air-dried in Petri dishes to obtain the nanocomposite.

## 3. Results and Discussion

### 3.1. Characterization of Medicago Sativa Extract

The extract obtained exhibited a dark brown coloration, with a total volume of 450 mL of concentrated solution. The dark coloration suggests the extraction of soluble compounds from alfalfa leaves. Bioactive compounds, such as polyphenols, flavonoids, and other secondary metabolites are characteristic of alfalfa extracts and potentially responsible for their reducing activity in the green hydrothermal synthesis of NPs [65]. These bioactive compounds function as reducing agents by donating electrons or hydrogen atoms to the metal ions. In the process of NP synthesis, metal ions (such as Cu or Co) are first reduced by these bioactive compounds, facilitating their transformation from metal salts to metal NPs [66].

**Figure 3:**
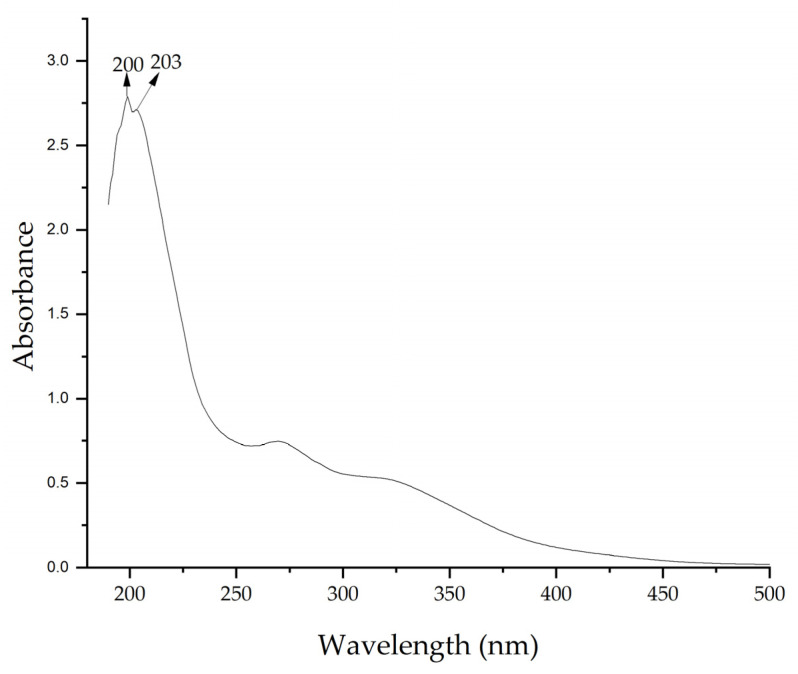
UV–vis spectrum of *Medicago sativa* extract.

UV–vis spectra of the extract confirmed the presence of bioactive compounds. Figure 3 shows a maximum absorption peak at 200 nm. These peaks indicate the presence of compounds with π bonds, such as those found in aromatic compounds or with conjugated double bonds, which are common in flavonoids and other phenolic compounds present in plant extracts [67,68]. The presence of these compounds allows the formation of NPs by acting as reducing agents.

**Figure 4:**
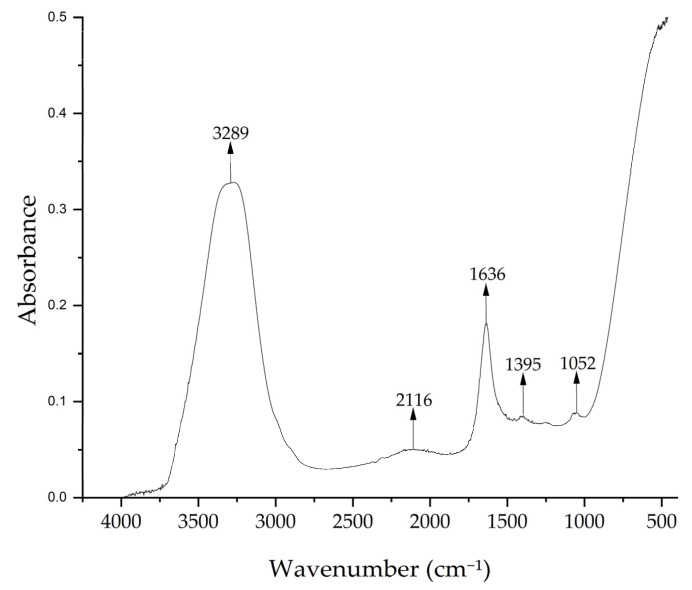
FTIR spectrum of *Medicago sativa* extract.

Figure 4 shows the FTIR spectrum of the *Medicago sativa* extract with peaks at 3289 cm^−1^, which indicates the presence of –OH groups, at 2116 cm^−1^ corresponding to C–H of hydro-carbon, and at 1636 cm^−1^ indicating the C=O of carbonyl bond, a band characteristic of the biologically active compounds such as flavonoids and polyphenols [50,51]. The peak at 1395 cm^−1^ could correspond to C–O stretching vibrations and C–C stretching vibrations of phenyl groups and the peak at 1052 cm^−1^ indicates the presence of C–O stretching vibrations, possibly from mono-, oligo-, and carbohydrates [69,70]. 

### 3.2. Characterization of NPs

The Cu-NPs obtained using solvothermal synthesis appeared as a fine dark gray powder with a yield of 32.5%, indicating efficient conversion under the reaction conditions employed (180 °C for 16 h). In contrast, the green hydrothermal synthesis, which utilized alfalfa extract as a reducing and stabilizing agent, produced NPs in the form of an intense green powder. This synthesis achieved a lower yield of 24.5%, probably due to the lower reducing power of the chemical components present in the plant extract.

On the other hand, the solvothermal synthesis of Co-NPs produced a dark reddish-brown powder, representing a yield of 26.7%, and the green hydrothermal synthesis produced NPs of dark brown to black powder, with a yield of 14.3%. The difference in yields may be due to ethylene glycol being a much more efficient reducing agent than the compounds present in the alfalfa extract.

**Figure 5:**
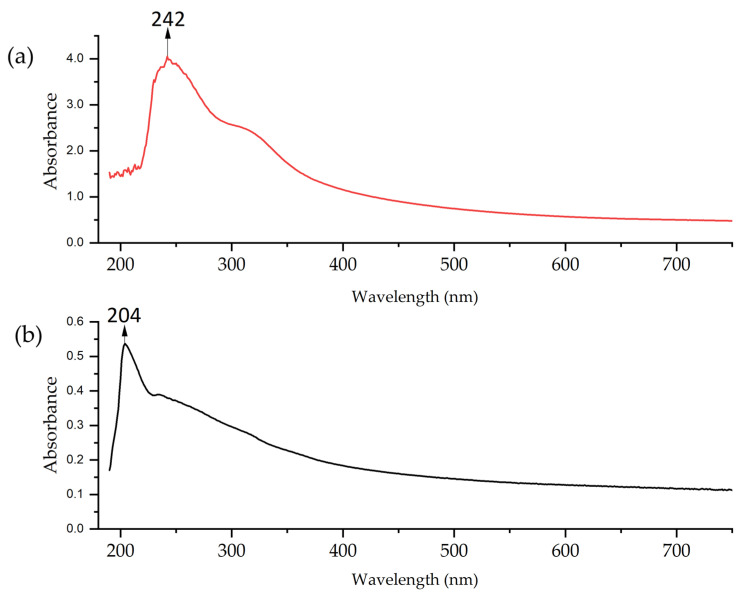
UV–vis of Cu-NPs: (**a**) solvothermal synthesis and (**b**) green hydrothermal synthesis.

The UV–vis analysis of Cu-NPs (Figure 5) revealed differences between both synthesis methods. The two curves show strong absorption in the UV region (200–440 nm), which gradually decreases towards longer wavelengths. The solvothermal NPs exhibited higher absorption in the 240–250 nm range. This suggests the predominant formation of oxide primarily [70]. On the other hand, the spectrum of Cu-NPs synthesized via the green hydrothermal synthesis showed lower and more uniform absorption, with a peak in the 200–240 nm range. This indicates either a greater dispersion of particle sizes or the potential presence of organic compounds from the extract in the NPs, with influence on the shift of the resonant wavelength, the width of the absorbance band, and even the appearance of a new absorption band [71].

**Figure 6:**
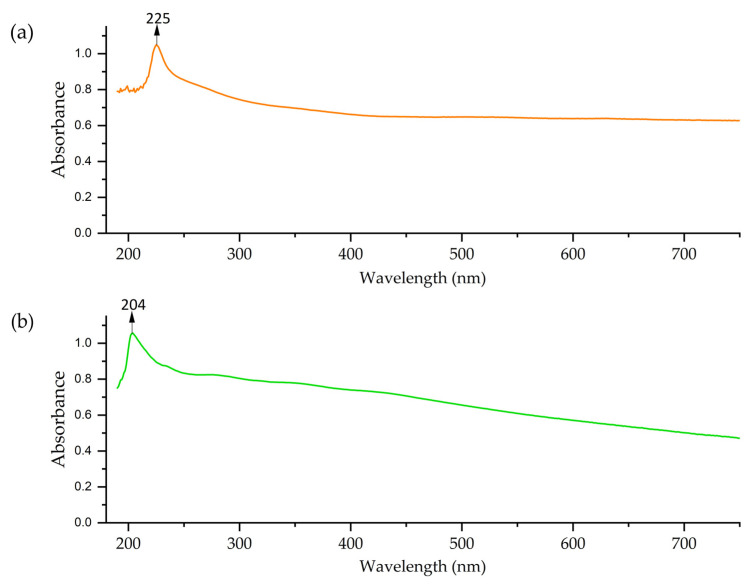
UV–vis of Co-NPs: (**a**) solvothermal synthesis and (**b**) green hydrothermal synthesis.

The UV–vis spectra of Co-NPs similarly showed different profiles. The solvothermal NPs presented an absorption peak at 230–240 nm, followed by a rapid decrease in absorption (Figure 6). This profile is consistent with the formation of Co-NPs; possibly a mixture of oxides [72]. The Co-NPs synthesized via green hydrothermal synthesis exhibited lower and constant absorption across the entire spectral range, without peaks. This absence of defined spectral features indicates the presence of organic residues from the alfalfa extract that modifies the optical properties of the NPs [73].

### 3.3. Determination of Point of Zero Charge

**Figure 7:**
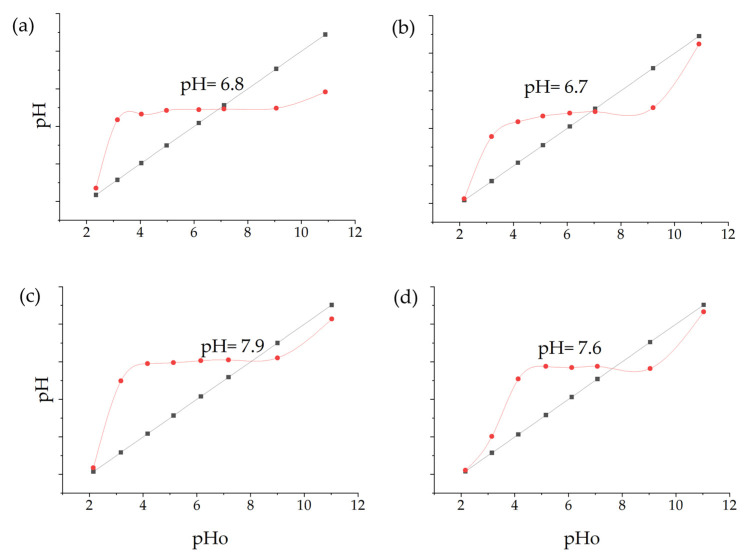
pH_pzc_: (**a**) Cu-NPs’ solvothermal synthesis; (**b**) Cu-NPs’ green hydrothermal synthesis; (**c**) Co-NPs’ solvothermal synthesis; (**d**) Co-NPs’ green hydrothermal synthesis (The black line represents the theoretical pH values, and the red line shows the actual measured pH values after the nanoparticles have interacted with the solution).

A pH_pzc_ of 6.8 was determined for Cu-NPs synthesized via the solvothermal method. The curve for these NPs started near pH 2, increased rapidly to pH 6–7, then experienced a slight increase and remained stable (Figure 7a). The Cu-NPs synthesized by the green hydrothermal synthesis had a pH_pzc_ of 6.7. Figure 7b shows a curve with complex behavior. It starts near pH 2, rises rapidly to pH 6, remains stable until pH 7, then descends slightly and rises again at the end. It can be determined that the pH_pzc_ was close to neutrality in both types of NPs, which is common for metal oxides [74].

On the other hand, the pH_pzc_ of Co-NPs synthesized by solvothermal methodology presented a curve that began near a pH of 2, increased rapidly to a pH of 7–8, and then followed a more gradual trend, having a pH_pzc_ value of 7.9 (Figure 7c). The Cu-NPs synthesized by green hydrothermal methodology presented a pH_pzc_ of 7.6, with a curve that rose rapidly to a pH of 7 (Figure 7d). Likewise, the obtained values are common in oxide NPs [75].

### 3.4. Morphological and Structural Analysis

**Figure 8:**
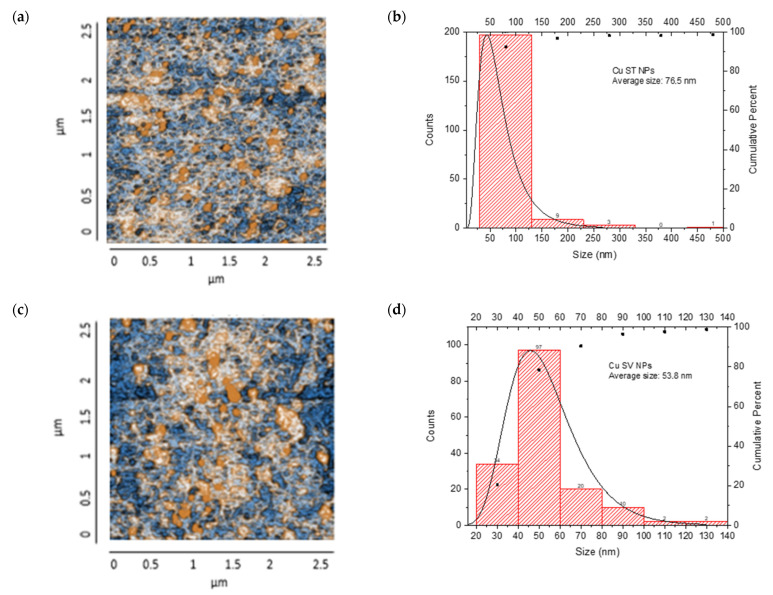

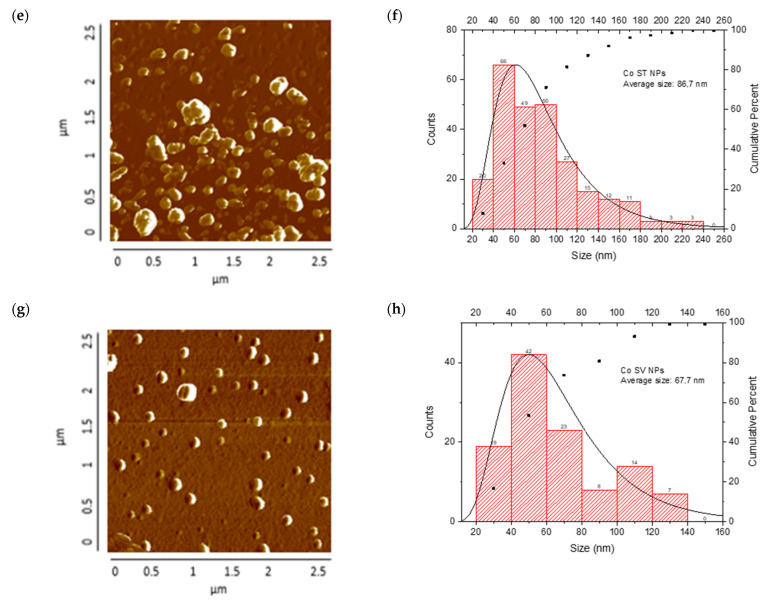
AFM analysis of NPs synthesized and particle size distribution: (**a**,**b**) Cu-NPs synthetized by solvothermal synthesis and (**c**,**d**) green hydrothermal synthesis. Co-NPs synthesized by (**e**,**f**) solvothermal synthesis and (**g**,**h**) green hydrothermal synthesis. The red histograms on the X-axis represent the size ranges of NPs expressed in nm and the quantity of NPs per range. The black line identifies the trend of NPs in relation to their quantity across sizes.

The AFM analysis of Cu-NPs obtained through solvothermal synthesis showed a homogeneous surface distribution of NPs with predominantly spherical morphology, although some agglomeration regions were observed. Size distribution analysis demonstrated a mean diameter of 76.5 nm. From the analyzed sample, most NPs were concentrated in the 1–100 nm range, with a smaller population at 100–200 nm, confirming that the solvothermal synthesis provided effective control over the size and morphology of Cu-NPs. The cumulative distribution curve indicated that approximately 95% of the particles were below 100 nm, as is shown in Figure 8a,b.

On the other hand, Figure 8c,d show Cu-NPs synthesized by the green hydrothermal method. The analysis showed a highly uniform surface distribution with predominantly spherical morphology, displaying notable dispersion and minimal agglomeration. The mean particle diameter was 53.8 nm, with a narrower and more symmetric distribution indicating better control of the synthesis process. The primary population was concentrated in the 40–60 nm range, with a significant secondary population at 60–80 nm. The cumulative distribution curve revealed that approximately 81% of the particles were below 100 nm, demonstrating that green hydrothermal synthesis provides an efficient and environmentally friendly method for obtaining Cu-NPs with well-defined morphological and dimensional characteristics.

Co-NPs synthesized by the solvothermal method presented a surface distribution with a tendency toward agglomeration and irregular morphology. Statistical analysis of the size distribution showed a mean diameter of 86.8 nm, where the main population was concentrated in the 40–60 nm range, followed by a significant secondary population at 80–100 nm. The presence of minor populations extending to 240 nm suggests less precise control over particle growth during nucleation and growth processes. The cumulative distribution showed that approximately 71% of the particles were below 100 nm (Figure 8e,f).

Finally, Figure 8g,h show Co-NPs synthesized by the green hydrothermal method. They showed an ordered surface distribution with predominantly spherical morphology and less tendency toward agglomeration compared to conventional methods. Statistical analysis of the size distribution revealed a mean diameter of 67.7 nm, showing a more homogeneous distribution with a main population in the 40–60 nm range, followed by a secondary population at 60–80 nm. The narrower distribution and absence of particles above 140 nm suggest that green hydrothermal synthesis provided better control over Co-NPs’ growth, resulting in a more uniform and defined population.

Green hydrothermal synthesis proved to be more effective in producing smaller NPs with a more uniform distribution, for both Cu and Co. This result is consistent with research suggesting that green hydrothermal synthesis can offer better control over the size and morphology of NPs due to the presence of natural stabilizing agents [76]. Although the NPs obtained by solvothermal synthesis were larger, they presented a higher density on the analyzed surface. This could be attributed to more controlled reaction conditions, allowing for faster particle growth [77]. It is important to note that the size and shape of NPs can significantly influence their physical and chemical properties, and therefore, their effectiveness as antifungal agents. The smaller and more uniform particles obtained by green hydrothermal synthesis could offer advantages in terms of cellular penetration [78].

### 3.5. FTIR Analysis

**Figure 9:**
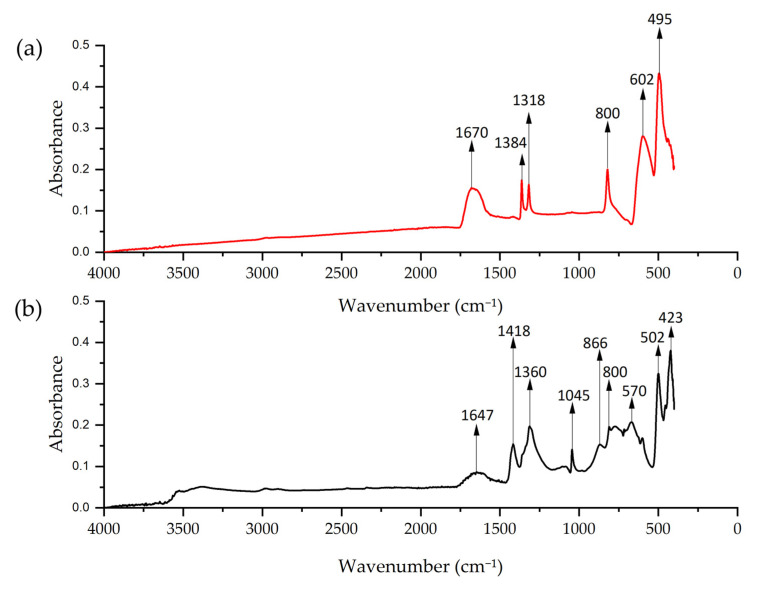
FTIR spectrum of Cu-NPs: (**a**) solvothermal synthesis NP and (**b**) green hydrothermal NPs.

FTIR analysis of Cu-NPs synthesized by solvothermal method showed a pronounced peak at 1670 cm^−1^ that could correspond to the O–H [79], possibly from ethylene glycol. The peaks at 1318 and 1384 cm^−1^ could correspond to the C=O stretching vibrations [80]. The peaks at 800, 602, and 495 cm^−1^ correspond to Cu–O vibrations [81,82,83]. For the green hydrothermal synthesis, the peak at 1647 cm^−1^ could correspond to C=O stretching, the peak at 1418 cm^−1^ could be C=C stretching, and the peak at 1360 cm^−1^ could correspond to aldehydic C-H stretching, suggesting the presence of flavonoids [84]. The peak at 1045 cm^−1^ is assigned to C–O stretching vibrations from carbohydrates, consistent with the alfalfa extract analysis [69]. The spectrum showed peaks at 866, 800, 570, 502, and 423 cm^−1^ that could correspond to characteristic vibration of Cu–O [85] (Figure 9). It was established that NPs synthesized by the green hydrothermal methodology presented more defined and complex peaks, suggesting the presence of organic compounds from the plant extract. Meanwhile, Cu-NPs obtained by solvothermal synthesis showed a smoother spectrum, with fewer defined peaks, indicating a simpler composition.

**Figure 10:**
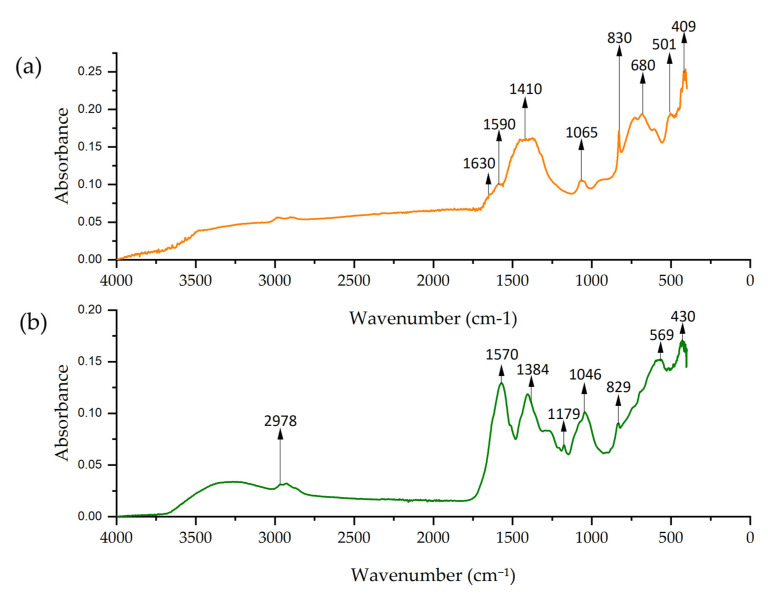
FTIR spectrum of Co-NPs: (**a**) solvothermal synthesis NPs and (**b**) green hydrothermal NPs.

FTIR spectra of Co-NPs obtained by solvothermal and green hydrothermal synthesis are presented in Figure 10. The solvothermal spectrum presents a peak at 1630 cm^−1^, which corresponds to the bending vibration of adsorbed water (H–O–H) [86]. At 1590 cm^−1^, the peak is attributed to the C=O stretching vibrations. The peaks at 1410, 1065, and 830 cm^−1^ formed a band that can be attributed to Co–O–H stretching [87].

Meanwhile, the spectrum of Co-NPs obtained by green hydrothermal synthesis showed a peak at 2978 cm^−1^ corresponding to the C–H stretching vibrations of the alkyl groups, probably from the organic compounds in the alfalfa extract [69]. The peak at 1384 cm^−1^ is assigned to C–H bending vibrations from organic compounds in the extract, and the peak at 1570 cm^−1^ could indicate the presence of C=C groups from the biomolecules [88]. Both spectra showed intense peaks at 680, 502, 409, and 569, 430 cm^−1^ corresponding to metal–oxygen (Co–O) vibrations [89].

### 3.6. Characterization of CS/Metal NP Nanocomposite by AFM

Each nanocomposite presented the same consistency; however, they possessed a characteristic color according to the NPs used. Four nanocomposites of CS/metal-NPs were obtained: CS/Cu-NPs (solvothermal), CS/Cu-NPs (green hydrothermal), CS/Co-NPs (solvothermal), and CS/Co-NPs (green hydrothermal).

**Figure 11:**
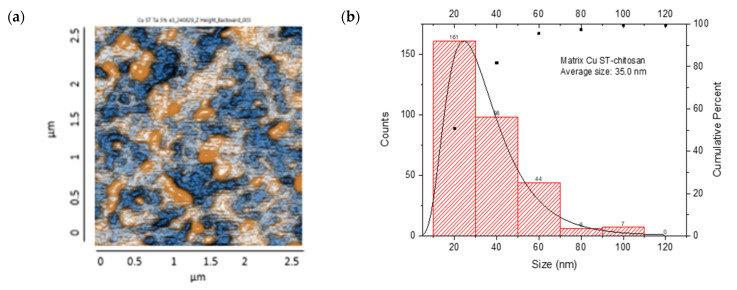

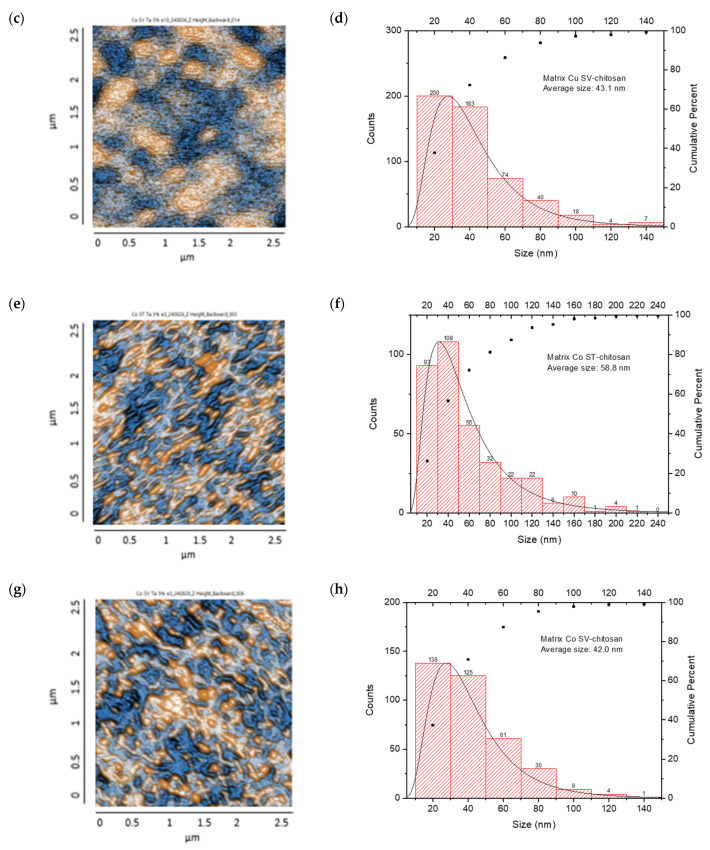
AFM images: CS/metal-NPs and particle size distribution: (**a**,**b**) Cu-NPs (solvothermal); (**c**,**d**) Cu-NPs (green hydrothermal); (**e**,**f**) Co-NPs (solvothermal); (**g**,**h**) Co-NPs (green hydrothermal). The red histograms on the X-axis represent the size ranges of NPs expressed in nm and the quantity of NPs per range. The black line identifies the trend of NPs in relation to their quantity across sizes.

The resulting nanocomposites showed an irregular surface with granular structures, with protrusions (orange color) and depressions (blue color) observed, suggesting a distribution of NP aggregates. The CS/Cu-NP (solvothermal) nanocomposite presented a surface with a uniform distribution of spherical Cu-NPs in the CS, with grains of an average length of 35.0 nm, suggesting a smooth surface with few irregularities (Figure 11a,b). The size distribution ranged from 20 to 120 nm, with a main population in the 20–40 nm range. The cumulative percentage curve demonstrated that approximately 65% of the NPs fell below 40 nm in diameter, indicating excellent size control during the synthesis process.

The CS/Cu-NP (green hydrothermal) nanocomposite had an average grain size of 43.1 nm in length, which indicated a surface with a few irregularities where the NPs had been adequately distributed in the CS (Figure 11c,d). The histogram exhibited a right-skewed distribution, with the highest frequency of particles occurring in the 20–40 nm range. The cumulative distribution curve indicated that approximately 91% of the particles were smaller than 80 nm, demonstrating effective size control through the green hydrothermal synthesis approach [90]. CS/Cu-NPs presented a uniform distribution of structures, which could lead to a controlled and sustained release of Cu ions, desirable for antifungal application [91].

On the other hand, the CS/Co-NP nanocomposite obtained by solvothermal synthesis showed a non-uniform particle distribution with elongated structures, with grains presenting an average length of 58.8 nm (Figure 11e,f). The histogram analysis revealed that most Co-NPs were concentrated in the 20–40 nm range, with a secondary population at 40–60 nm, and diminishing frequencies for larger sizes up to 240 nm. The cumulative distribution curve indicated that approximately 97% of the particles fell below 100 nm in size.

The CS/Co-NP (green hydrothermal) nanocomposite presented a more homogeneous distribution in which agglomerations and rounded structures were observed, and the grains revealed an average length of 42 nm (Figure 11g,h). The histogram data showed the highest frequency population in the 20–40 nm range, followed by a secondary population at 40–60 nm, and progressively smaller populations extending to 140 nm. The cumulative distribution curve indicated that approximately 91% of the particles were below 80 nm, suggesting that the green hydrothermal synthesis approach combined with CS stabilization effectively controlled particle growth and agglomeration.

AFM analysis of the generated nanocomposites revealed that combinations of CS/metal-NPs (solvothermal) presented a larger grain size than matrices with green hydrothermal NPs; therefore, the surfaces of these turned out to be rougher [92].

### 3.7. SEM Characterization (CS/Metal NP Nanocomposites)

**Figure 12:**
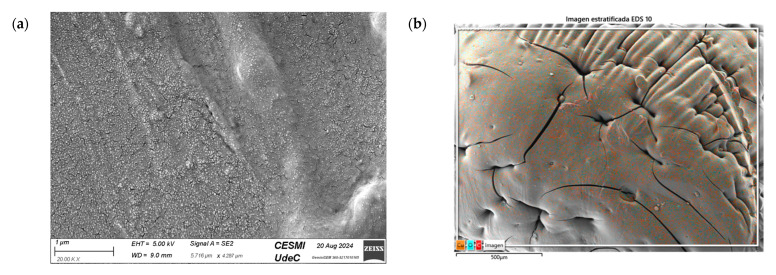

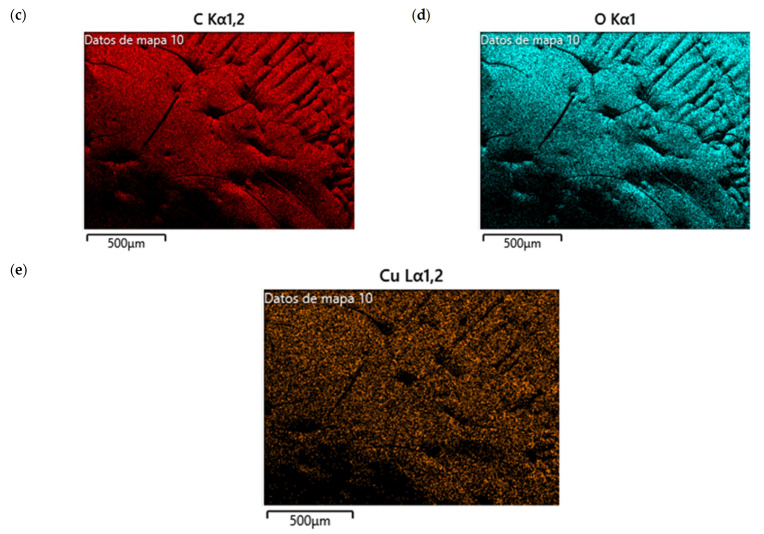
SEM of CS/Cu-NP (green hydrothermal) nanocomposites: (**a**) image with 20.00 KX magnification; (**b**) EDS stratified image; (**c**) map data for C; (**d**) map data for O; (**e**) map data for Cu.

The SEM analysis in Figure 12a reveals a rough and heterogeneous surface texture. The surface morphology suggests a successful incorporation of the NPs in CS matrix. Carbon is also highlighted in the sample, indicating the presence of CS (Figure 12b,c), and the presence of Cu (Figure 12d), demonstrating that the nanocomposite contained Cu-NPs in its composition.

**Figure 13:**
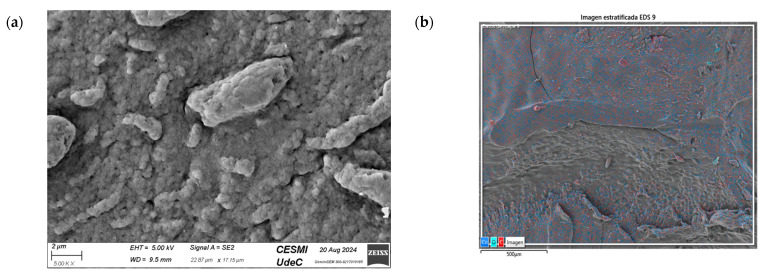

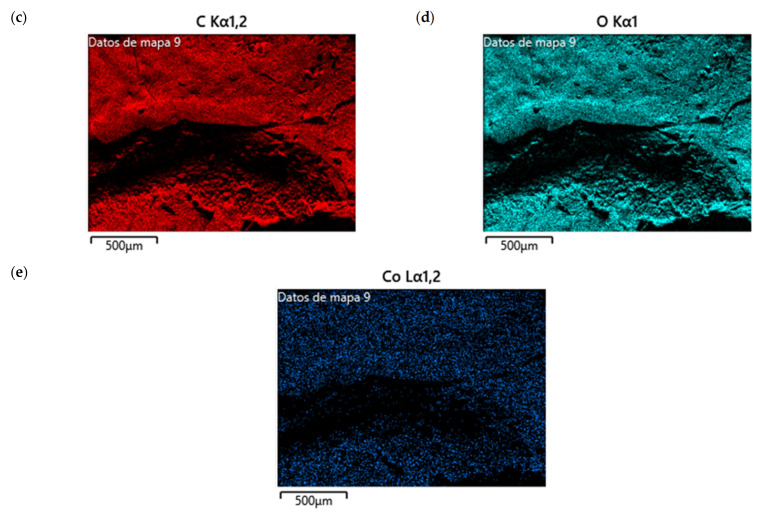
SEM of CS/Co-NP (green hydrothermal) nanocomposite: (**a**) image with 5.00 KX magnification; (**b**) EDS stratified image; (**c**) map data for C; (**d**) map data for O; (**e**) map data for Co.

Figure 13a shows the SEM images of CS/Co-NPs (green hydrothermal). The analysis revealed a complex surface where the Co-NPs appeared as aggregates distributed throughout the sample. According to Figure 13c, the nanocomposite contained the presence of C in its structure due to CS. Figure 13e shows the presence of Co was evident throughout the surface of nanocomposite.

### 3.8. Efficiency of Antifungal or Inhibitory Action

Bioassays were conducted using the synthesized NPs and the *Pseudocercospora fijiensis* fungus. After 30 days of incubation, notable differences were observed in the growth of the *Pseudocercospora fijiensis* between control treatments and those with NPs. In all bioassays, the total cultivation area was 63.61 cm^2^. The images of the cultures were analyzed in their real colors and in 8-bit format, in which the images are displayed in black and white. In the 8-bit format, the area occupied by the *Pseudocercospora fijiensis* is highlighted in red (Table 1 and Table 2).

### 3.9. Bioassays with NPs (Solvothermal)

The control plate exhibited the highest fungal growth, with dark and dense colonies covering a large part of the culture medium. Both treatments with Cu-NPs and Co-NPs showed no fungal growth at the dose used, with the same small and sparse colonies observed as on day 0 (Table 1). The control culture for solvothermal NPs exhibited a mycelial growth area of 23.78 cm^2^, representing 37.36% of the total area. The plate containing Cu-NPs showed an initial coverage area of 1.85 cm^2^ (2.91%), corresponding to fungal growth. Similarly, the plate with Co-NPs presented an initial coverage area of 1.10 cm^2^ (1.72%). After 30 days of *Pseudocercospora fijiensis* incubation, both treatments with Cu-NPs and Co-NPs showed no mycelial growth at the dose used, as the fungus maintained the same occupied area.

The results suggest that both Co-NPs and Cu-NPs had an inhibitory effect on the growth of the *Pseudocercospora fijiensis*. This effect could be attributed to the antimicrobial properties of Cu, which have been reported in previous studies [49,93]. Cu-NPs and Co-NPs may interfere with essential metabolic processes of the fungus or directly damage its cellular structures [94,95].

### 3.10. Bioassays with NPs (Green Hydrothermal)

After the growth period, the control plate showed significant fungal growth, with dark and dense colonies covering a large part of the medium. In the plate with Co-NPs, it was observed that the medium maintained its deep brown color and there was no growth compared to the control. In the plate with Cu-NPs, the medium was observed with the initial light green hydrothermal color and similarly, there was no fungal growth (Table 2). The control culture for NPs (green hydrothermal) exhibited a coverage area of 19.82 cm^2^, equivalent to 31.15%. The plate containing Cu-NPs showed an initial coverage area of 1.01 cm^2^ (1.58%), corresponding to fungal growth. Similarly, the plate with Co-NPs presented an initial coverage area of 1.12 cm^2^ (1.76%). After 30 days of *Pseudocercospora fijiensis* incubation, no mycelial growth was observed.

Both Cu-NPs and Co-NPs synthesized by the green hydrothermal method showed inhibitory effects on the growth of the *Pseudocercospora fijiensis* fungus at the dose used.

## 4. Conclusions

This comparative study demonstrated that solvothermal synthesis using ethylene glycol produced higher NP yields than green hydrothermal synthesis with *Medicago sativa* extract. However, the green hydrothermal approach resulted in smaller (53.8 nm for Cu-NPs and 67.7 nm for Co-NPs, respectively) and more uniform NPs with less agglomeration compared to the solvothermal approach (76.5 nm for Cu-NPs and 86.8 nm for Co-NPs, respectively). The FTIR analysis showed that the organic compounds from the alfalfa extract persisted after the synthesis of NPs. AFM revealed that NPs (solvothermal) had larger grains and rougher surfaces than green hydrothermal synthesis. SEM characterization of nanocomposite confirmed the successful incorporation of NPs in the CS matrix. These results suggest that green hydrothermal synthesis represents a promising and sustainable approach for producing well-defined metallic nanostructures with controlled size distributions. Both solvothermal and green hydrothermal NPs exhibited complete inhibition of the target fungus *Pseudocercospora fijiensis* at 2000 ppm. Thus, the use of green hydrothermal synthesis to produce Cu-NPs and Co-NPs proves to be a promising and potentially more environmentally friendly alternative for the control of phytopathogenic fungi.

## Figures and Tables

**Table 1:** Bioassays of NPs synthesized by solvothermal methodology after 30 days of fungal incubation.

	Control Plate	Cu-NPs	Co-NPs
True color image (RGB)	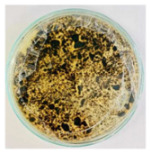	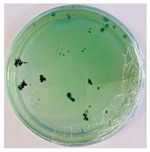	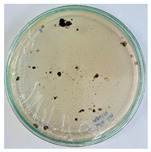
False color image (8 bits)	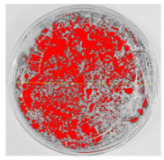	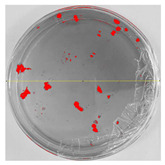	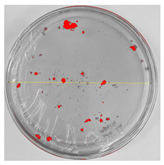

**Table 2:** Bioassays of NPs synthesized by green hydrothermal synthesis after 30 days of fungal incubation.

	Control Plate	Cu-NPs	Co-NPs
True color image (RGB)	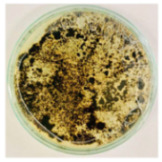	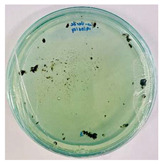	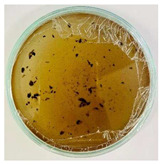
False color image (8 bits)	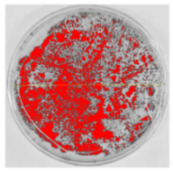	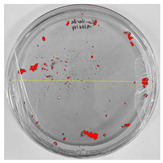	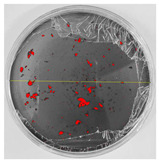

## Data Availability

The original contributions presented in the study are included in the article, further inquiries can be directed to the corresponding author.

## Abbreviations

The following abbreviations are used in this manuscript:

NPs	nanoparticles
CS	chitosan
UV–vis	UV–visible spectroscopy
FTIR	Fourier-transform infrared spectroscopy
AFM	atomic force microscopy
SEM	scanning electron microscope
EDS	energy dispersive spectrometry
FE-SEM	field emission scanning electron microscopy
PDA	potato dextrose agar
PZC	point of zero charge
pH_pzc_	pH of the point of zero charge
NCHR	non-contact/high-resonance frequency reflex coating

## References

[1] Labanni A., Nasir M., Arief S. (2023). Research progress and prospect of copper oxide nanoparticles with controllable nanostructure, morphology, and function via green synthesis. Mater. Today Sustain..

[2] Kaningini A.G., Nelwamondo A.M., Azizi S., Maaza M., Mohale K.C. (2022). Metal Nanoparticles in Agriculture: A Review of Possible Use. Coatings.

[3] Tomar R., Abdala A.A., Chaudhary R., Singh N. (2020). Photocatalytic degradation of dyes by nanomaterials. Mater. Today Proc..

[4] Roduner E. (2006). Size matters: Why nanomaterials are different. Chem. Soc. Rev..

[5] Asha A.B., Narain R., Na-rain R. (2020). Chapter 15—Nanomaterials properties. Polymer Science and Nanotechnology.

[6] Krishnan S.K., Singh E., Singh P., Meyyappan M., Singh Nalwa H. (2019). A review on graphene-based nanocomposites for electrochemical and fluorescent biosensors. RSC Adv..

[7] Mittal A.K., Banerjee U.C., Grumezescu A.M. (2016). Chapter 5—Current status and future prospects of nanobiomaterials in drug delivery. Nanobiomaterials in Drug Delivery.

[8] Wu Q., Miao W.-S., Zhang Y.-D., Gao H.-J., Hui D. (2020). Mechanical properties of nanomaterials: A review. Nanotechnol. Rev..

[9] Vijayaprasath G., Murugan R., Mahalingam T., Ravi G. (2015). Comparative study of structural and magnetic properties of transition metal (Co, Ni) doped ZnO nanoparticles. J. Mater. Sci. Mater. Electron..

[10] Cruz-Luna A.R., Cruz-Martínez H., Vásquez-López A., Medina D.I. (2021). Metal Nanoparticles as Novel Antifungal Agents for Sustainable Agriculture: Current Advances and Future Directions. J. Fungi.

[11] Issa B., Obaidat I.M., Albiss B.A., Haik Y. (2013). Magnetic nanoparticles: Surface effects and properties related to biomedicine applications. Int. J. Mol. Sci..

[12] Babu G.A., Ravi G. (2016). Magnetic evolution in transition metal-doped Co3−xMxO4 (M = Ni, Fe, Mg and Zn) nanostructures. Appl. Phys. A.

[13] Hu Y., Ji C., Wang X., Huo J., Liu Q., Song Y. (2017). The structural, magnetic and optical properties of TMn@(ZnO)42 (TM = Fe, Co and Ni) hetero-nanostructure. Sci. Rep..

[14] Jawoor S.S., Patil S.A., Kumbar M., Ramawadgi P.B. (2018). Green synthesis of nano sized transition metal complexes containing heterocyclic Schiff base: Structural and morphology characterization and bioactivity study. J. Mol. Struct..

[15] Azam A., Ahmed A.S., Oves M., Khan M.S., Habib S.S., Memic A. (2012). Antimicrobial activity of metal oxide nanoparticles against Gram-positive and Gram-negative bacteria: A comparative study. Int. J. Nanomed..

[16] Atarod M., Nasrollahzadeh M., Sajadi S.M. (2015). Green synthesis of a Cu/reduced graphene oxide/Fe_3_O_4_ nanocomposite using *Euphorbia wallichii* leaf extract and its application as a recyclable and heterogeneous catalyst for the reduction of 4-nitrophenol and rhodamine B. RSC Adv..

[17] Nasrollahzadeh M., Maham M., Sajadi S.M. (2015). Green synthesis of CuO nanoparticles by aqueous extract of Gundelia tournefortii and evaluation of their catalytic activity for the synthesis of N -monosubstituted ureas and reduction of 4-nitrophenol. J. Colloid Interface Sci..

[18] Li Y., Liang J., Tao Z., Chen J. (2008). CuO particles and plates: Synthesis and gas-sensor application. Mater. Res. Bull..

[19] Yuhas B.D., Yang P. (2009). Nanowire-Based All-Oxide Solar Cells. J. Am. Chem. Soc..

[20] Lee B.H., Ng M.Z., Zinn A.A., Gan C.L. Application of copper nanoparticles as die attachment for high power LED. Proceedings of the 2015 IEEE 17th Electronics Packaging and Technology Conference (EPTC).

[21] Luechinger N.A., Athanassiou E.K., Stark W.J. (2008). Graphene-stabilized copper nanoparticles as an air-stable substitute for silver and gold in low-cost ink-jet printable electronics. Nanotechnology.

[22] Dong H., Meininger A., Jiang H., Moon K.-S., Wong C.P. (2007). Magnetic Nanocomposite for Potential Ultrahigh Frequency Microelectronic Application. J. Electron. Mater..

[23] Iravani S., Varma R.S. (2020). Sustainable synthesis of cobalt and cobalt oxide nanoparticles and their catalytic and biomedical applications. Green Chem..

[24] Pourmadadi M., Holghoomi R., Shamsabadipour A., Maleki-Baladi R., Rahdar A., Pandey S. (2024). Copper nanoparticles from chemical, physical, and green synthesis to medicinal application: A review. Plant Nano Biol..

[25] Gebreslassie Y.T., Gebremeskel F.G. (2024). Green and cost-effective biofabrication of copper oxide nanoparticles: Exploring antimicrobial and anticancer applications. Biotechnol. Rep..

[26] López-Moreno M.L., Avilés L.L., Pérez N.G., Irizarry B.Á., Perales O., Cedeno-Mattei Y., Román F. (2016). Effect of cobalt ferrite (CoFe2O4) nanoparticles on the growth and development of Lycopersicon lycopersicum (tomato plants). Sci. Total. Environ..

[27] Sharma P., Sharma A., Sharma M., Bhalla N., Estrela P., Jain A., Thakur P., Thakur A. (2017). Nanomaterial Fungicides: In Vitro and In Vivo Antimycotic Activity of Cobalt and Nickel Nanoferrites on Phytopathogenic Fungi. Glob. Challenges.

[28] Ogunyemi S.O., Xu X., Xu L., Abdallah Y., Rizwan M., Lv L., Ahmed T., Ali H.M., Khan F., Yan C. (2023). Cobalt oxide nanoparticles: An effective growth promoter of Arabidopsis plants and nano-pesticide against bacterial leaf blight pathogen in rice. Ecotoxicol. Environ. Saf..

[29] Shaikh A.J., Rasheed R., Tahir M.B., Bakhat H.F., Rafique M.S., Rabbani F. (2017). A Review on Synthesis, Characterization and Applications of Copper Nanoparticles Using Green Method. Nano.

[30] Gahlawat G., Choudhury A.R. (2019). A review on the biosynthesis of metal and metal salt nanoparticles by microbes. RSC Adv..

[31] Saleh T.A. (2020). Nanomaterials: Classification, properties, and environmental toxicities. Environ. Technol. Innov..

[32] Devadoss D., Asirvatham A., Kujur A., Saaron G., Devi N., Mary S.J. (2023). Green synthesis of copper oxide nanoparticles from Murraya koenigii and its corrosion resistivity on Ti-6Al-4V dental alloy. J. Mech. Behav. Biomed. Mater..

[33] Nyabadza A., McCarthy É., Makhesana M., Heidarinassab S., Plouze A., Vazquez M., Brabazon D. (2023). A review of physical, chemical and biological synthesis methods of bimetallic nanoparticles and applications in sensing, water treatment, biomedicine, catalysis and hydrogen storage. Adv. Colloid Interface Sci..

[34] Sánchez D.B., Hubenthal F., Träger F. (2007). Shaping nanoparticles with laser light: A multi-step approach to produce nanoparticle ensembles with narrow shape and size distributions. J. Phys. Conf. Ser..

[35] Kumar P., Krishna M.G. (2010). A comparative study of laser- and electric-field-induced effects on the crystallinity, surface morphology and plasmon resonance of indium and gold thin films. Phys. Status Solidi (a).

[36] Mescola A., Canale C., Fragouli D., Athanassiou A. (2017). Controlled formation of gold nanostructures on biopolymer films upon electromagnetic radiation. Nanotechnology.

[37] Balu S.K., Andra S., Jeevanandam J., Kulabhusan P.K., Khamari A., Vedarathinam V., Hamimed S., Chan Y.S., Danquah M.K. (2023). Exploring the potential of metal oxide nanoparticles as fungicides and plant nutrient boosters. Crop. Prot..

[38] Guzmán Barba T., Estrada Flores M., Rojas Valencia O.G., Manríquez Ramírez M.E., Reza San Germán C.M. (2019). Síntesis Mediante la Técnica Solvotermal de Partículas Nanoestructuradas de WO_3_ Dopadas con S Palabras Clave.

[39] Lai J., Niu W., Luque R., Xu G. (2015). Solvothermal synthesis of metal nanocrystals and their applications. Nano Today.

[40] Satyavani T., Kumar A.S., Rao P.S. (2016). Methods of synthesis and performance improvement of lithium iron phosphate for high rate Li-ion batteries: A review. Eng. Sci. Technol. Int. J..

[41] Wen S., Bao G., Jin D., Yin Y., Lu Y., Xia Y. (2023). Advanced optical properties of upconversion nanoparticles. Encyclopedia of Nano-Materials.

[42] Chatterjee S., Mahanty S., Das P., Chaudhuri P., Das S. (2020). Biofabrication of iron oxide nanoparticles using manglicolous fungus Aspergillus niger BSC-1 and removal of Cr(VI) from aqueous solution. Chem. Eng. J..

[43] Nath D., Banerjee P. (2013). Green nanotechnology—A new hope for medical biology. Environ. Toxicol. Pharmacol..

[44] Shedbalkar U., Singh R., Wadhwani S., Gaidhani S., Chopade B. (2014). Microbial synthesis of gold nanoparticles: Current status and future prospects. Adv. Colloid Interface Sci..

[45] Shahzadi S., Fatima S., Ul Ain Q., Shafiq Z., Janjua M.R.S.A. (2025). A review on green synthesis of silver nanoparticles (SNPs) using plant extracts: A multifaceted approach in photocatalysis, environmental remediation, and biomedicine. RSC Adv..

[46] Dousari A.S., Hosseininasab S.S., Akbarizadeh M.R., Naderifar M., Satarzadeh N. (2023). Mentha pulegium as a source of green synthesis of nanoparticles with antibacterial, antifungal, anticancer, and antioxidant applications. Sci. Hortic..

[47] Iliger K.S., Sofi T.A., Bhat N.A., Ahanger F.A., Sekhar J.C., Elhendi A.Z., Al-Huqail A.A., Khan F. (2021). Copper nanoparticles: Green synthesis and managing fruit rot disease of chilli caused by Colletotrichum capsici. Saudi J. Biol. Sci..

[48] Vinayagam R., Hebbar A., Kumar P.S., Rangasamy G., Varadavenkatesan T., Murugesan G., Srivastava S., Goveas L.C., Kumar N.M., Selvaraj R. (2023). Green synthesized cobalt oxide nanoparticles with photocatalytic activity towards dye removal. Environ. Res..

[49] Pariona N., Mtz-Enriquez A.I., Sánchez-Rangel D., Carrión G., Paraguay-Delgado F., Rosas-Saito G. (2019). Green-synthesized copper nanoparticles as a potential antifungal against plant pathogens. RSC Adv..

[50] Zare-Bidaki M., Aramjoo H., Mizwari Z.M., Mohammadparast-Tabas P., Javanshir R., Mortazavi-Derazkola S. (2022). Cytotoxicity, antifungal, antioxidant, antibacterial and photodegradation potential of silver nanoparticles mediated via Medicago sativa extract. Arab. J. Chem..

[51] Król A., Railean-Plugaru V., Pomastowski P., Buszewski B. (2019). Phytochemical investigation of Medicago sativa L. extract and its potential as a safe source for the synthesis of ZnO nanoparticles: The proposed mechanism of formation and antimicrobial activity. Phytochem. Lett..

[52] Mulu M., RamaDevi D., Belachew N., Basavaiah K. (2021). Hydrothermal green synthesis of MoS_2_ nanosheets for pollution abatement and antifungal applications. RSC Adv..

[53] Ocsoy I., Demirbas A., McLamore E.S., Altinsoy B., Ildiz N., Baldemir A. (2017). Green synthesis with incorporated hydrothermal approaches for silver nanoparticles formation and enhanced antimicrobial activity against bacterial and fungal pathogens. J. Mol. Liq..

[54] Yaghoobi M., Asjadi F., Sanikhani M. (2023). A facile one-step green hydrothermal synthesis of paramagnetic Fe3O4 nanoparticles with highly efficient dye removal. J. Taiwan Inst. Chem. Eng..

[55] Tiwari A.D., Mishra A.K., Mishra S.B., Kuvarega A.T., Mamba B.B. (2013). Stabilisation of silver and copper nanoparticles in a chemically modified chitosan matrix. Carbohydr. Polym..

[56] de Godoi F.C., Rabelo R.B., Silva M.A., Rodríguez-Castellón E., Guibal E., Beppu M.M. (2014). Introduction of copper nanoparticles in chitosan matrix as strategy to enhance chromate adsorption. Chem. Eng. Process.-Process. Intensif..

[57] Lincoln S., Chowdhury P., Posen P.E., Robin R., Ramachandran P., Ajith N., Harrod O., Hoehn D., Harrod R., Townhill B.L. (2023). Interaction of climate change and marine pollution in Southern India: Implications for coastal zone management practices and policies. Sci. Total. Environ..

[58] Bebber D.P. (2019). Climate change effects on Black Sigatoka disease of banana. Philos. Trans. R. Soc. B Biol. Sci..

[59] de C. Exterior M. (2017). Informe Sector Bananero. https://www.produccion.gob.ec/wp-content/uploads/2019/06/Informe-sector-bananero-espa%C3%B1ol-04dic17.pdf.

[60] Álvarez S.P., López N.E.L., Lozano J.M., Negrete E.A.R., Cervantes M.E.S., Prasad R. (2016). Plant Fungal Disease Manage-ment Using Nanobiotechnology as a Tool. Advances and Applications Through Fungal Nanobiotechnology.

[61] Marcia H., Sánchez G., Aguilar E.E.J., Noemi S., Reyes H., Aguilar J. (2022). Fungicides based on sulfur and bacillus sp. in integrated ma-nagement of black sigatoka. Rev. Científica Agroecosistemas.

[62] Cerna-Chávez E., Malacara-Herrera I.D.R., Ochoa-Fuentes Y.M., Hernández-Juárez A. (2023). Evaluación in vitro de extractos vegetales adicionados con nanopartículas para el control de Fusarium oxysporum. Ecosistemas Recur. Agropecu..

[63] Jindal K. (2023). Synthesis, Characterization and Evaluation of Antifungal activity of Copper Nanoparticle. https://krishikosh.egranth.ac.in/handle/1/5810209194.

[64] Vera-Reyes I., Altamirano-Hernández J., la Cruz H.R.-D., Granados-Echegoyen C.A., Loera-Alvarado G., López-López A., Garcia-Cerda L.A., Loera-Alvarado E. (2022). Inhibition of Phytopathogenic and Beneficial Fungi Applying Silver Nanoparticles In Vitro. Molecules.

[65] Song K., Zhao D., Sun H., Gao J., Li S., Hu T., He X. (2022). Green nanopriming: Responses of alfalfa (*Medicago sativa* L.) seedlings to alfalfa extracts capped and light-induced silver nanoparticles. BMC Plant Biol..

[66] Marslin G., Siram K., Maqbool Q., Selvakesavan R.K., Kruszka D., Kachlicki P., Franklin G. (2018). Secondary Metabolites in the Green Synthesis of Metallic Nanoparticles. Materials.

[67] Patle T.K., Shrivas K., Kurrey R., Upadhyay S., Jangde R., Chauhan R. (2020). Phytochemical screening and determination of phenolics and flavonoids in Dillenia pentagyna using UV–vis and FTIR spectroscopy. Spectrochim. Acta. A. Mol. Biomol. Spectrosc..

[68] Alhakmani F., Alam Khan S., Ahmad A. (2014). Determination of total phenol, in-vitro antioxidant and anti-inflammatory activity of seeds and fruits of Zizyphus spina-christi grown in Oman. Asian Pac. J. Trop. Biomed..

[69] Caunii A., Pribac G., Grozea I., Gaitin D., Samfira I. (2012). Design of optimal solvent for extraction of bio–active ingredients from six varieties of Medicago sativa. BMC Chem..

[70] Khalaji A.D., Pazhand Z., Kiani K., Machek P., Jarosova M., Mazandarani R. (2020). CuO nanoparticles: Preparation, characterization, optical properties, and antibacterial activities. J. Mater. Sci. Mater. Electron..

[71] Jayarambabu N., Akshaykranth A., Rao T.V., Rao K.V., Kumar R.R. (2020). Green synthesis of Cu nanoparticles using Curcuma longa extract and their application in antimicrobial activity. Mater. Lett..

[72] Farhadi S., Javanmard M., Nadri G. (2016). Characterization of Cobalt Oxide Nanoparticles Prepared by the Thermal Decomposition of [Co(NH3)5(H2O)](NO3)3 Complex and Study of Their Photocatalytic Activity. Acta Chim. Slov..

[73] Rónavári A., Igaz N., Adamecz D.I., Szerencsés B., Molnar C., Kónya Z., Pfeiffer I., Kiricsi M. (2021). Green Silver and Gold Nanoparticles: Biological Synthesis Approaches and Potentials for Biomedical Applications. Molecules.

[74] Cristiano E., Hu Y.-J., Sigfried M., Kaplan D., Nitsche H. (2011). A Comparison of Point of Zero Charge Measurement Methodology. Clays Clay Miner..

[75] Kosmulski M. (2018). The pH dependent surface charging and points of zero charge. VII. Update. Adv. Colloid Interface Sci..

[76] Mostafazade R., Arabi L., Tazik Z., Akaberi M., Bazzaz B.S.F. (2024). Green synthesis of gold, copper, zinc, iron, and other metal nanoparticles by fungal endophytes; characterization, and their biological activity: A review. Biocatal. Agric. Biotechnol..

[77] Ramakrishnan V.M., Li J., Wu Q., Wu J., Aliofkhazraei M. (2015). Synthesis of Nanoparticles via Solvothermal and Hydrothermal Methods. Handbook of Nanoparticles.

[78] Castañeda M.T. (2019). Enzimas de Interés Biotecnológico. http://sedici.unlp.edu.ar/handle/10915/89649.

[79] Mullaivendhan J., Akbar I., Ahamed A., Alodaini H.A. (2024). Synthesis rifaximin with copper (Rif-Cu) and copper oxide (Rif-CuO) nanoparticles Considerable dye decolorization: An application of aerobic oxidation of eco-friendly sustainable approach. Heliyon.

[80] Kayani Z.N., Umer M., Riaz S., Naseem S. (2015). Characterization of Copper Oxide Nanoparticles Fabricated by the Sol–Gel Method. J. Electron. Mater..

[81] Sahai A., Goswami N., Kaushik S., Tripathi S. (2016). Cu/Cu2O/CuO nanoparticles: Novel synthesis by exploding wire technique and extensive characterization. Appl. Surf. Sci..

[82] Swarnkar R.K., Singh S.C., Gopal R. (2011). Effect of aging on copper nanoparticles synthesized by pulsed laser ablation in water: Structural and optical characterizations. Bull. Mater. Sci..

[83] Khulbe R., Kumar R., Kumar V., Kandpal A., Joshi R., Chandra B., Kandpal N.D. (2024). Synthesis and Characterization of Copper Oxide Nanoparticles Using Polyol and Their Antimicrobial Potential. Mater. Int..

[84] Nagar N., Devra V. (2018). Green synthesis and characterization of copper nanoparticles using Azadirachta indica leaves. Mater. Chem. Phys..

[85] Hassanien R., Husein D.Z., Al-Hakkani M.F. (2018). Biosynthesis of copper nanoparticles using aqueous Tilia extract: Antimicrobial and anticancer activities. Heliyon.

[86] Hutamaningtyas E., Utari, Suharyana, Wijayanta A.T., Purnama B. (2016). FTIR and structural properties of co-precipitated cobalt ferrite nano particles. J. Phys. Conf. Ser..

[87] Arora S., Singla M.L., Kapoor P. (2009). Evidence for monoalkoxide species on the surface of palladium nanoparticles synthesized in ethylene glycol. Mater. Chem. Phys..

[88] Slavov D., Tomaszewska E., Grobelny J., Drenchev N., Karashanova D., Peshev Z., Bliznakova I. (2024). FTIR spectroscopy revealed nonplanar conformers, chain order, and packaging density in diOctadecylamine- and octadecylamine-passivated gold nanoparticles. J. Mol. Struct..

[89] Nallusamy S., Sujatha K. (2021). Experimental analysis of nanoparticles with cobalt oxide synthesized by coprecipitation method on electrochemical biosensor using FTIR and TEM. Mater. Today Proc..

[90] Maldonado-Lara K., Luna-Bárcenas G., Luna-Hernández E., Padilla-Vaca F., Hernández-Sánchez E., Betancourt-Galindo R., Menchaca-Arredondo J.L., España-Sánchez B.L. (2017). Preparación y Caracterización de Nanocompositos Quitosano-Cobre con Actividad Antibacteriana para aplicaciones en Ingeniería de Tejidos. Rev. Mex. Ing. Bioméd..

[91] Silva A.O., Cunha R.S., Hotza D., Machado R.A.F. (2021). Chitosan as a matrix of nanocomposites: A review on nanostructures, processes, properties, and applications. Carbohydr. Polym..

[92] Lamarra J., Giannuzzi L., Rivero S., Pinotti A. (2017). Assembly of chitosan support matrix with gallic acid-functionalized nanoparticles. Mater. Sci. Eng. C.

[93] Lopez-Lima D., Mtz-Enriquez A.I., Carrión G., Basurto-Cereceda S., Pariona N. (2021). The bifunctional role of copper nanoparticles in tomato: Effective treatment for Fusarium wilt and plant growth promoter. Sci. Hortic..

[94] Barros J., Kumar S., Seena S. (2023). Does functionalised nanoplastics modulate the cellular and physiological responses of aquatic fungi to metals?. Environ. Pollut..

[95] Wang L., Hu C., Shao L. (2017). The antimicrobial activity of nanoparticles: Present situation and prospects for the future. Int. J. Nanomed..

