# Correction: Caguana et al. Metal Nanoparticles Obtained by Green Hydrothermal and Solvothermal Synthesis: Characterization, Biopolymer Incorporation, and Antifungal Evaluation Against *Pseudocercospora fijiensis*. *Nanomaterials* 2025, *15*, 379

**DOI:** 10.3390/nano16040229

**Published:** 2026-02-10

**Authors:** Tania Caguana, Christian Cruzat, David Herrera, Denisse Peña, Valeria Arévalo, Mayra Vera, Pablo Chong, Néstor Novoa, Ramón Arrué, Eulalia Vanegas

**Affiliations:** 1 Master Program in Environmental Sciences, Faculty of Chemical Sciences, Eco Campus Balzay, University of Cuenca, Cuenca 010207, Ecuador; tania.caguana@ucuenca.edu.ec; 2 N@NO-CEA Group, Center for Environmental Studies, Department of Applied Chemistry and Production Systems, Faculty of Chemical Sciences, University of Cuenca, Cuenca 010203, Ecuador; david.herrerar@ucuenca.edu.ec (D.H.); denisse.pena@ucuenca.edu.ec (D.P.); valeriaf.arevalo@ucuenca.edu.ec (V.A.); eulalia.vanegas@ucuenca.edu.ec (E.V.); 3 TECNO-CEA Group, Center for Environmental Studies, Department of Applied Chemistry and Production Systems, Faculty of Chemical Sciences, University of Cuenca, Cuenca 010203, Ecuador; mayra.vera@ucuenca.edu.ec; 4 Centro de Investigaciones Biotecnológicas del Ecuador, Laboratorio de Biología Molecular, Campus Gustavo Galindo, ESPOL Polytechnic University, Km 30.5 Vía Perimetral, Guayaquil 090902, Ecuador; pachong@espol.edu.ec; 5 Laboratorio de Química Inorgánica y Organometálica, Departamento de Química Analítica e Inorgánica, Facultad de Ciencias Químicas, Universidad de Concepción, Edmundo Larenas 129, Casilla 160-C, Concepción 4070386, Chile; nenovoa@udec.cl; 6 Facultad de Medicina y Ciencia, Departamento de Ciencias Biológicas y Químicas, Universidad San Sebastian, Lientur 1457, Concepción 4070386, Chile; ramon.arrue@uss.cl

In the original publication [1], the reference 45 has been retracted. The authors want to replace it with the following text, citation and reference:

The current phrase in our article: “Tesfaye et al. mention that active components such as flavonoids, saponins, alkaloids, tannins, and plant phenolics can reduce metal salts into stable NPs [45]” must be replaced for: “A recent and noteworthy review on the green synthesis of nanoparticles highlights the key role of active components—such as flavonoids, saponins, alkaloids, tannins, and plant phenolics—in reducing metal salts to stable nanoparticles [45].” Considering a new reference 45:

45. Shahzadi, S.; Fatima, S.; Ul Ain, Q.; Shafiq, Z.; Janjua, M.R.S.A. A review on green synthesis of silver nanoparticles (SNPs) using plant extracts: A multifaceted approach in photocatalysis, environmental remediation, and biomedicine. *RSC Adv.* **2025**, *15*, 3858–3903.

The authors state that the scientific conclusions are unaffected. This correction was approved by the Academic Editor. The original publication has also been updated.
